# Comparison of Different Approaches to Surface Functionalization of Biodegradable Polycaprolactone Scaffolds

**DOI:** 10.3390/nano9121769

**Published:** 2019-12-12

**Authors:** Elizaveta S. Permyakova, Philipp V. Kiryukhantsev-Korneev, Kristina Yu. Gudz, Anton S. Konopatsky, Josef Polčak, Irina Y. Zhitnyak, Natalia A. Gloushankova, D. V. Shtansky, Anton M. Manakhov

**Affiliations:** 1 National University of Science and Technology “MISIS”, Leninsky prospect 4, Moscow 119049, Russia; permyakova.elizaveta@gmail.com (E.S.P.); kiruhancev-korneev@yandex.ru (P.V.K.-K.); kristinkagudz@mail.ru (K.Y.G.); konopatskiy@misis.ru (A.S.K.); shtansky@shs.misis.ru (D.V.S.); 2 CEITEC—Central European Institute of Technology, Brno University of Technology, Purkyňova 123, 601 90 Brno, Czech Republic; polcak@fme.vutbr.cz; 3 Institute of Physical Engineering, Brno University of Technology, Technicka 2896/2, 616 69 Brno, Czech Republic; 4 N.N. Blokhin Russian Cancer Research Center, Kashirskoe shosse 24, Moscow 115478, Russia; irishaz@mail.ru (I.Y.Z.); natglu@hotmail.com (N.A.G.); 5 Scientific Institute of Clinical and Experimental Lymphology– Branch of the ICG SB RAS, 2 Timakova str., Novosibirsk 630060, Russia

**Keywords:** tissue engineering, polycaprolactone nanofibers, plasma modification, mineralization, XPS

## Abstract

Due to their good mechanical stability compared to gelatin, collagen or polyethylene glycol nanofibers and slow degradation rate, biodegradable poly-ε-caprolactone (PCL) nanofibers are promising material as scaffolds for bone and soft-tissue engineering. Here, PCL nanofibers were prepared by the electrospinning method and then subjected to surface functionalization aimed at improving their biocompatibility and bioactivity. For surface modification, two approaches were used: (i) COOH-containing polymer was deposited on the PCL surface using atmospheric pressure plasma copolymerization of CO_2_ and C_2_H_4_, and (ii) PCL nanofibers were coated with multifunctional bioactive nanostructured TiCaPCON film by magnetron sputtering of TiC–CaO–Ti_3_PO_x_ target. To evaluate bone regeneration ability in vitro, the surface-modified PCL nanofibers were immersed in simulated body fluid (SBF, 1×) for 21 days. The results obtained indicate different osteoblastic and epithelial cell response depending on the modification method. The TiCaPCON-coated PCL nanofibers exhibited enhanced adhesion and proliferation of MC3T3-E1 cells, promoted the formation of Ca-based mineralized layer in SBF and, therefore, can be considered as promising material for bone tissue regeneration. The PCL–COOH nanofibers demonstrated improved adhesion and proliferation of IAR-2 cells, which shows their high potential for skin reparation and wound dressing.

## 1. Introduction

Tissue engineering is one of the most important areas of modern medicine aimed at healing or replacement of damaged tissues and organs by age, disease, or trauma [1]. Depending on their function in the body, implants should be replaced gradually with a living tissue and/or service for a long time. One of the problems in the field of tissue engineering that is not solved yet is to develop scaffolds that mimic the architecture of tissue at the nanoscale. Bone tissue consists of different types of bone cells (osteoblasts, osteocytes, and osteoclasts) and a mineralization matrix formed by collagen nanofibers and nano-hydroxyapatite (nano-HA) [2]. Since the nanofiber structure is similar to the extracellular matrix, which is an important controller of cell adhesion, proliferation, migration, and differentiation, nanofiber is a promising structural element for scaffold fabrication [3,4,5]. Due to its simplicity and good reproducibility, electrospinning is a widely used method for the production of polymer, ceramics, and metal nanofibers. Since the nature of polymer affects tissue regeneration and drug release kinetics, different types of precursors were used in the electrospinning process [6]. Among various biopolymers of particular interest is poly(ε-caprolactone) (PCL), which is a biodegradable polyester due to its susceptibility to hydrolytic cleavage of the ester bond [7]. Moreover, PCL has good mechanical properties and has been approved by the American Food and Drug Administration (FDA) for biomedical applications. Currently, it is used as part of the suture (Monocryl, EthiconEndo-Surgery, Inc.) and endodontic dental (Resilon) materials [8]. The shortcoming of PCL, as well as many other polymers, include their hydrophobicity and bioinertness that prevent cell attachment and, therefore, do not provide interfacial bonding. In contrast, the bioactive surface initiates the precipitation and growth of HA crystallites which act as an intermediate binder layer between the implant and bone tissue [9,10].

To avoid time-consuming and expensive in vivo experiments, the material bioactivity, as a first approximation, can be evaluated in vitro. Immersion of biomaterials in the simulated body fluid (SBF), which is a solution with ion concentration close to that of human blood plasma, and further study of the mechanisms and kinetics of bone-like apatite formation is a widely used method [11]. In a number of studies, more concentrated SBF was utilized. For example, Zhang et al. [12] used five times SBF (5× SBF) to cause rapid mineral deposition on the surface of poly L-lactide PLLA/gelatin composite nanofibers to avoid possible nanofiber degradation during biomineralization. The highly supersaturated 5× SBF was continuously bubbled with carbon dioxide to keep the solution transparent throughout the experiment. Nagarajan et al. [13] carried out biomineralization of gelatin nanofibers using 1.5× SBF. Boron nitride (BN) nanoparticles added to the gelatin nanofibers were shown to significantly increase their bioactivity since the vacant “p” orbital of the boron atom in the BN is responsible for the Lewis acid nature. Gao et al. [14] also used SBF to cover poly (L-lactic-co-glycolic acid)-tussah silk fibroin nanofiber by HA layer. Moreover, the authors demonstrated that the compressive modulus and stress of the mineralized composite scaffolds were 32.8 and 3.0 times higher, respectively than those of the composite scaffolds without mineralization. 

An alternative method of soaking mineralization was proposed by Taguchi et al. [15]. Scaffolds were repeatedly soaked in calcium-rich (0.5 M CaCl_2_) and phosphate-rich (0.3 M Na_2_HPO_4_) solutions (usually no more than 10 times) to form a HA layer on their surface. This allows one to increase the CaP nucleation rate and ensure the formation of stable CaP precipitates in less than 1 min. The same approach was employed by Wei et al. [16] to study the mineralization of silk fibroin. The primary nano-HA crystals with a diameter approximately 30 nm were observed on the surface of nanofibers after 3 cycles. 

Electrolysis using the scaffold three-electrode system with a constant voltage is another method that has been successfully utilized for nanofiber mineralization [17]. To deposit Ca_3_(PO_4_)_2_ on the surface of a sample, all electrodes (scaffold is fixed on the cathode) are placed in an electrolyte (NH_4_H_2_PO_4_, Ca(NO_3_)_2_, pH 4.7) and the electrochemical station is used for electrodeposition. After deposition, the electrodes are removed from the electrolyte and rinsed with distilled water. 

Other approaches to enhance material bioactivity are to add nano-HA into electrospun solution or to deposit amorphous calcium phosphate (ACP) on the nanofiber surface. The deposition of ACP is achieved by sequential soaking in solutions of calcium chloride and sodium phosphate [18]. 

To be suitable materials for bone fillers or wound healing, the surface of PCL-based scaffolds should be factionalized to provide desired bioactive characteristics. Previous studies suggested that the wettability of a polymer surface can be improved by the deposition of hydrophilic film [19]. Unlike high-energy ion irradiation of polymers, which can lead to its destruction and toxicity [20], magnetron sputtering with carefully optimized processing parameters is an effective method to coat biodegradable polymer with highly adhesive biocompatible film [21]. Another promising approach for the PCL surface modification is COOH plasma polymerization. In the present study, two approaches to endow PCL with enhance bioactivity were used and compared: (i) atmospheric pressure plasma copolymerization of CO_2_ and C_2_H_4_ to form COOH-containing polymer and (ii) magnetron sputtering of TiC–CaO–Ti_3_PO_x_ target to deposit TiCaPCON film. [21]. Another promising approach for the PCL surface modification is COOH plasma polymerization. Previously, TiCaPCON coatings were deposited on the surface of metals, insoluble polymers, and deimmunized donor’s bone [22]. Coated metal implants have successfully passed clinical trials and, therefore, were selected for the final PCL surface modification The bioactivity of surface-modified PCL was studied in SBF. After the SBF tests, the samples were characterized in term of their structure and surface chemical states using a combination of various microanalytical techniques, such as scanning electron microscopy (SEM), Fourier transform infrared (FTIR) spectroscopy, and X-ray photoelectron spectroscopy (XPS). Proliferation tests using two types of cells, namely MC3T3 osteoblastic and IAR-2 epithelial cells, were carried out to evaluate possible scaffold applications as bone fillers or wound dressing. 

## 2. Materials and Methods

### 2.1. Electrospinning of Poly-ε-Caprolactone (PCL) Nanofibers

PCL polymer (Sigma Aldrich, Germany) with a molecular weight of approximately 80,000 g/mol in amount of 9 wt.% was dissolved in a 2:1 mixture of acetic acid (99%, Sigma Aldrich, Germany) and formic acid (98%, Sigma Aldrich, Germany). The electrospinning process was carried out on a Nanospider™ NSLAB 500 (ELMARCO, Czech Republic) machine using a 20 cm-long wired electrode at a voltage of 55 kV according to the protocol available elsewhere [21]. The distance between high voltage and ground electrodes was set at 100 mm. Further information about the electrospinning process can be found elsewhere [21]. Spinning PCL samples were denoted as PCL-ref. The thickness of the PCL scaffold was ~100 µm.

### 2.2. Deposition of TiCaPCON Film

TiCaPCON film (hereafter designated as PCL–TiCaPCON) was deposited onto the surface of PCL nanofibers by magnetron sputtering of a composite TiC–CaO–Ti_3_PO_x_ target produced by the self-propagating high-temperature synthesis. Deposition experiments were carried out on a “UNICOAT 900” unit under the following process parameters: target to substrate distance—200 mm, accelerating voltage—450 V, deposition time—10 min, Ar (99.995 %) and N_2_ (99.999%) flow rates—250 and 25 sccm, respectively. 

### 2.3. Deposition of COOH Plasma Polymers

The deposition of COOH-rich plasma polymers on to the surface of PCL nanofibers (samples denoted as PCL–COOH) was carried out using a vacuum system, UVN-2M, equipped with rotary and oil diffusion pumps providing the residual pressure in a vacuum chamber below 10^−3^ Pa. Ar (99.998%), CO_2_ (99.995%), and C_2_H_4_ (99.95%) gases were used as precursors. More details about the PCL plasma polymerization can be found elsewhere [23]. 

### 2.4. Biomineralization of PCL Nanofibrous Scaffolds

SBF tests were carried out to study sample biomineralization using the standard protocol as described elsewhere [24]. The PCL-ref, PCL–COOH, and PCL–TiCaPCON samples were cut into plates, 1 × 1 cm^2^, and immersed in SBF solution for 1, 3, 7, and 14 days. Every 24 h, the SBF solution was replaced with a new one.

### 2.5. Material Characterization

The scaffold morphology was examined by SEM and atomic force microscopy (AFM). SEM analysis was carried out with a JSMF 7600 microscope (JEOL Ltd.) equipped with an energy-dispersive X-ray spectrometer. To compensate for surface charge, the samples were coated with a ~5 nm thick Pt layer. The average fiber diameter was determined based on size measurements of at least 50 fibers using SEM images. The mean value and standard deviation for elemental concentrations and fiber diameters were calculated using MS Excel. AFM analysis of electrospun nanofiber mats was performed on an Integra Spectra microscope (NT-MDT) using silicon-nitride NSG10 scanning probes (NT-MDT) with a tip radius of 8 ± 2.1 nm. 

The chemical characterization of samples was performed by XPS, energy-dispersive X-ray spectroscopy (EDXS) and FTIR spectroscopy. FTIR spectra (100 scans) were recorded in increments of 4 cm^−1^ on a Vertex 80v FTIR spectrophotometer (Bruker) with a parallel beam transmittance accessory. The spectra were collected at room temperature (20–25 °C). The surface chemical composition was determined by the XPS using an Axis Supra spectrometer (Kratos Analytical). The maximum lateral dimension of the analyzed area was 0.7 mm. The spectra were fitted using CasaXPS software after subtracting Shirley-type background. The binding energy (BE) values for all carbon, titanium, and nitrogen environments were taken from the available literature [23,25,26,27]. The BE scale was calibrated by shifting CH_x_ component to 285.0 eV. 

The sample wettability was assessed by measuring the water contact angle (WCA). The measurements were carried out on an Easy Drop Kruss (KRṺSS, Germany) device. For each sample at least five WCA measurements were performed.

### 2.6. Morphometric Analysis

To test biocompatibility, the mouse osteoblastic MC3T3-E1 (ATCC) and normal epithelial rat IAR-2 cells (International Agency for Research on Cancer [28]) were used. The IAR-2 cell line is the immortalized epithelial cell line with normal phenotype that is frequently used as a model cell culture. MC3T3-E1 (10^4^ cells/mL) and IAR (10^4^ cells/mL) cells were seeded on the surface of samples placed into 12-well plates containing DMEM/F12 culture medium (Gibco), in case of MC3T3-E1, or DMEM (Sigma), in case of IAR, with 10% of fetal calf serum (PAA Laboratories). 

The cultures were grown in a humidified atmosphere with 5% of CO_2_ at 37 °C for 24 h. Then the cells were fixed with 3.7% paraformaldehyde (Sigma) for 10 min, permeated with 0.5% Triton X-100 (Sigma), and stained with primary antibodies against paxillin (Becton Dickinson). The incubation was further continued after washing with phosphate-buffered saline (PBS) three times and adding TRITC-conjugated goat anti-mouse IgG (Jackson ImmunoResearch) and Alexa488-phalloidin (Molecular Probes). 

Twenty images of single cells from the fluorescent staining experiments were acquired in the green channel of a Nikon Eclipse Ti-E microscope equipped with a Plan Fluor 40× objective and ORCA-ER camera (Hamamatsu Photonics) controlled via NIS-Elements AR 3.22 software (Nikon). The mean area of cells was determined using ImageJ (LOCI, University of Wisconsin, US) software ver. 1.8.0. The Kruskal–Wallis test was used to compare differences between sample groups.

### 2.7. Actin Cytoskeleton and Focal Adhesions Staining

MC3T3-E1 and IAR-2 cells fixed by paraformaldehyde (PFA) were incubated with Alexa488-phalloidin and primary antibodies against paxillin (Becton Dickinson). The incubation was further continued after washing with PBS three times and adding TRITC-conjugated goat anti-mouse IgG (Jackson ImmunoResearch). Actin cytoskeleton and focal adhesions were observed using an Axioplan microscope.

### 2.8. Proliferation of MC3T3-E1 Osteoblastic and IAR-2 Epithelial Cells

MC3T3-E1 cells (10^4^ cells/mL) and IAR-2 cells (10^4^ cells/mL) were seeded on the surface of tested samples and cultivated in DMEM/F12 culture medium (MC3T3-E1) or DMEM (IAR-2) with 10% fetal bovine serum (FBS). After 1, 3, 5, and 7 days of incubation, cells were fixed with 3.7% paraformaldehyde (Sigma) and stained with 4′,6-diamidino-2-phenylindole (DAPI, Sigma). The number of cells in the field was determined with a Zeiss Axioplan microscope equipped with a Plan-Neofluar ×40 objective and a DP70 camera (Olympus). The means of thirty examined fields for each tested plate were calculated. Eaach samples group consisted of at least 5 samples prepared in the same batch. The Kruskal–Wallis test was used to compare differences between sample groups.

## 3. Results

### 3.1. Structural Characterization of PCL Nanofibers

**Figure 1:**
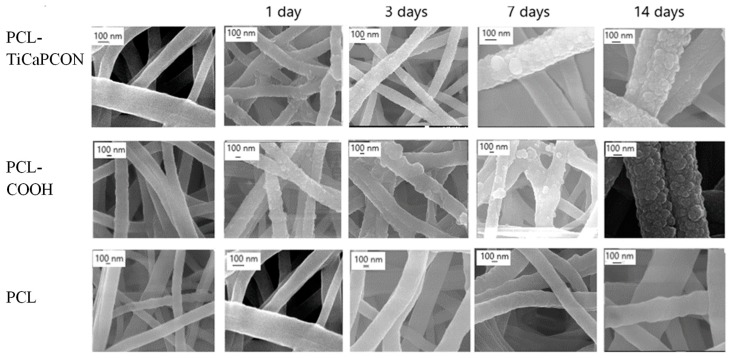
Scanning electron microscope (SEM) images of poly-ε-caprolactone (PCL)-ref, PCL–COOH, and PCL–TiCaPCON nanofibers before (left column) and after biomimetic mineralization for 1, 3, 7 and 14 days.

The morphology of as-prepared PCL-ref, PCL–COOH, and PCL–TiCaPCON samples is shown in Figure 1. The diameter of pristine nanofibers without surface modification determined from the SEM micrographs (n = 50) was 230 ± 50 nm, whereas the PCL–COOH and PCL–TiCaPCON samples exhibited the fibers with a diameter of 280 ± 40 and 270 ± 50 nm, respectively. Hence, the thickness increase after the deposition of thin COOH and TiCaPCON layers was very small. The nanofiber surface modification was further evidenced by thorough EDXS, WCA, and XPS studies. The EDXS analysis revealed Ti (~1.6 at.%), Ca (0.1 at.%), C (78.1 at.%) and O (19.8 at.%) in PCL–TiCaPCON nanofibers, whereas PCL-ref and PCL–COOH samples contained only C (~80 at.%) and O (~20 at.%). 

**Figure 2:**
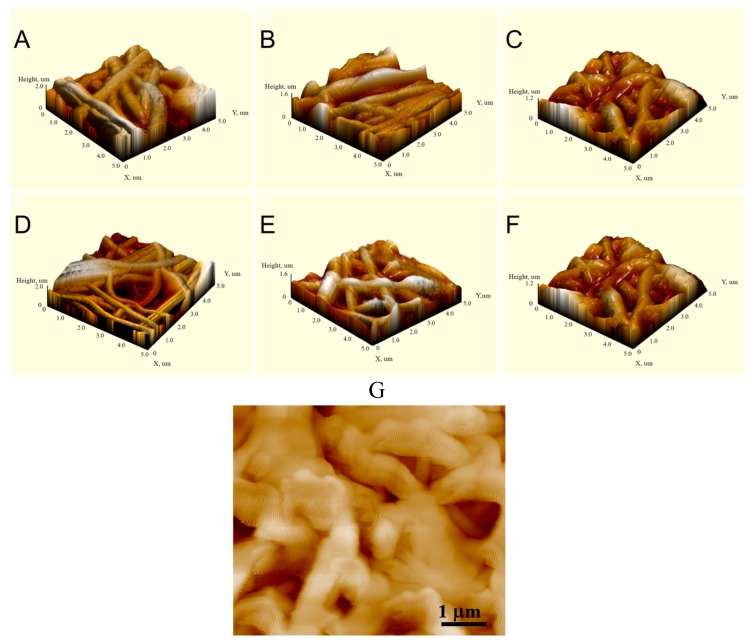
Atomic force microscopy (AFM) images of PCL-ref (**A**), PCL–TiCaPCON (**B**), PCL–COOH (**C**), PCL-ref-SBF-72h (**D**), PCL–COOH-SBF-72h (**E**), and PCL–TiCaPCON-SBF-72h (**F**,**G**) samples.

The AFM analysis (Figure 2) confirmed that after surface modifications, the structure and topography of the nanofibers were preserved. After the SBF tests, the AFM image of PCL-ref reveals clear nanofiber boundaries, whereas the surfaces of PCL–COOH and PCL–TiCaPCON samples look more blurry. Nanofibers become shapless and show characteristic wavy contrast due to being coated with a mineralazed layer. 

**Figure 3:**
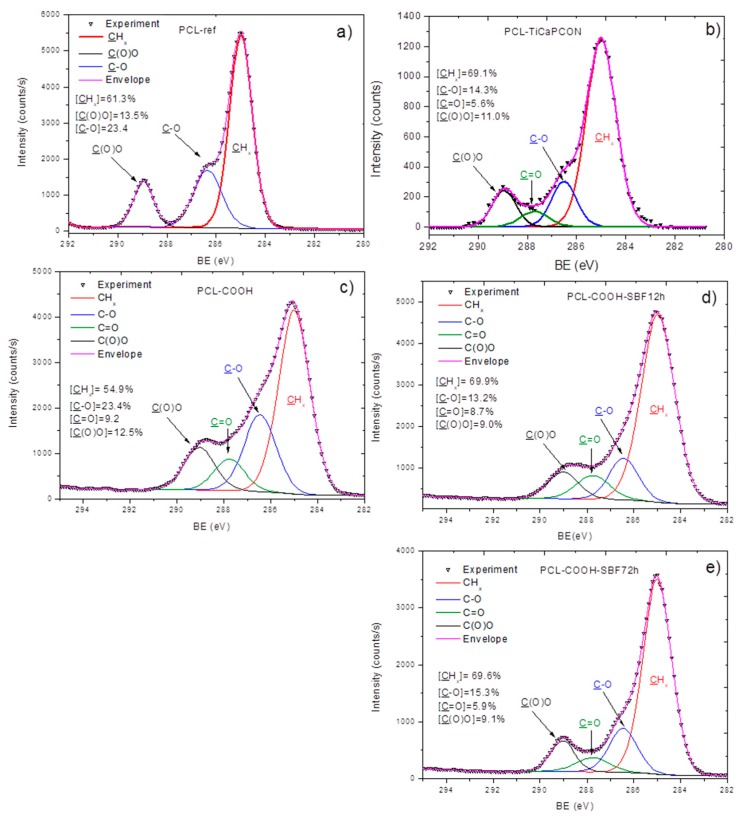
XPS C1s spectra of as-prepared nanofibers (**a**–**c**) and those after immersion in simulated body fluid (SBF) for 12 (**d**) and 72 h (**e**). PCL-ref (**a**), PCL–TiCaPCON (**b**), and PCL–COOH (**c**–**e**).

The XPS analysis demonstrated significant changes in the surface composition after deposition of TiCaPCON and COOH layers. The formation of TiCaPCON thin film was evidenced by the incorporation of titanium (~8.4 at.%), while the COOH plasma polymerization led to a small change in the O/C ratio. In addition, the XPS C1s spectra revealed significant changes in the carbon environment induced by the deposition of COOH plasma polymer. The XPS C1s spectrum of PCL-ref (Figure 3a) can be fitted with a sum of three components, namely hydrocarbons CH_x_ (BE = 285 eV), ether group C–O (BE = 286.4 eV), and ester group C(O)O (BE = 289.0 eV). The full width at the half maximum (FWHM) of C–O was set to 1.35 eV, while CH_x_ and C(O)O were fitted with the FWHM of 1.1 and 0.95 eV, respectively.

The XPS C1s spectra of PCL–TiCaPCON (Figure 3b) and PCL–COOH (Figure 3c) were fitted with four carbon components: **C**H_x_ (BE = 285 eV), **C**–O (BE = 286.45 ± 0.05 eV), **C**=O (BE = 287.8 ± 0.05 eV), and **C**(O)O (BE = 289.1 ± 0.05 eV). The FWHM value for all components was set to 1.4 ± 0.05 eV. The appearance of new environment and significant broadening of the peaks clearly indicated the changes in the surface composition. 

### 3.2. Water Contact Angle

**Figure 4:**
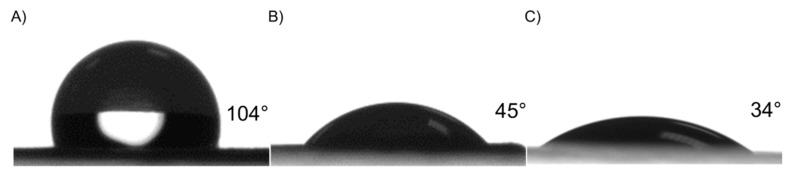
Water contact angle of PCL-ref (**A**), PCL–COOH (**B**) and PCL–TiCaPCON (**C**).

Plasma surface polymerization and TiCaPCON film deposition affected surface wettability (Figure 4). The PCL-ref sample exhibits hydrophobic characteristics, as the WCA is approximately 104 ± 3°. The deposition of Ar/CO_2_/C_2_H_4_ plasma layer led to a decrease in WCA to 45 ± 1.4°. After the deposition of TiCaPCON film, the WCA value was 34 ± 2°. Taking into account that the topography of PCL-ref, PCL–COOH and PCL–TiCaPCON samples were similar (Figure 1 and Figure 2), the wettability improvement of the PCL nanofibers can be explained by the grafting of polar groups [5]. The chemical composition of the PCL-ref sample, as determined by XPS, is reported in Table 1. 

### 3.3. Tests in Simulated Body Fluid (SBF)

**Figure 5:**
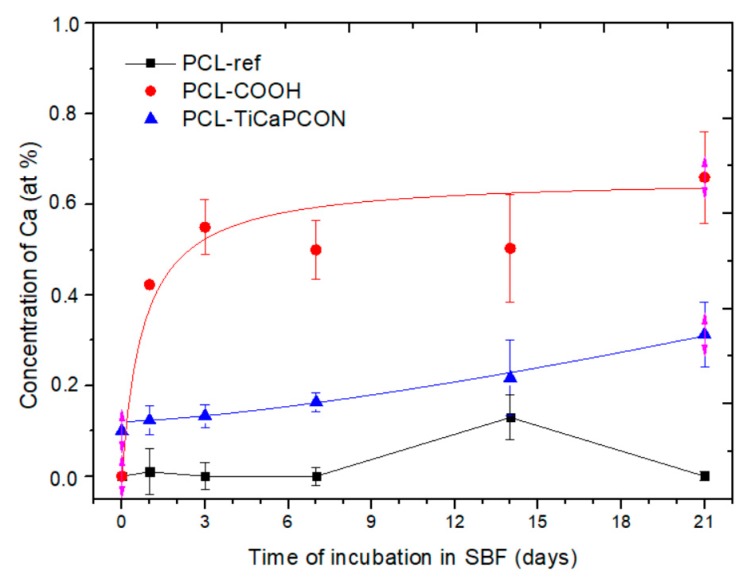
The concentration of Ca as a function of immersion time in SBF.

After mineralization experiments, PCL-ref, PCL–TiCaPCON, and PCL–COOH samples were analyzed by SEM, EDXS, FTIR spectroscopy, and XPS. SEM micrographs revealed that, after immersion in SBF for 24 h, the surface of PCL–COOH and PCL–TiCaPCON nanofibers were densely populated with spherical particles, 50–100 nm in diameter (Figure 1). After 14 days of mineralization, the size of mineral particles increased two times and they covered almost the entire nanofiber surface. The sample elemental compositions after the SBF tests were determined by EDXS. The change of Ca concentration over time is depicted in Figure 5. After immersion in SBF for 3 h, the Ca content in the PCL–COOH sample increased up to 0.6 at.% and did not changed during further exposure. In contrast, PCL-ref nanofibers immersed in SBF did not exhibit neither calcium nor nanoparticle formation. 

**Figure 6:**
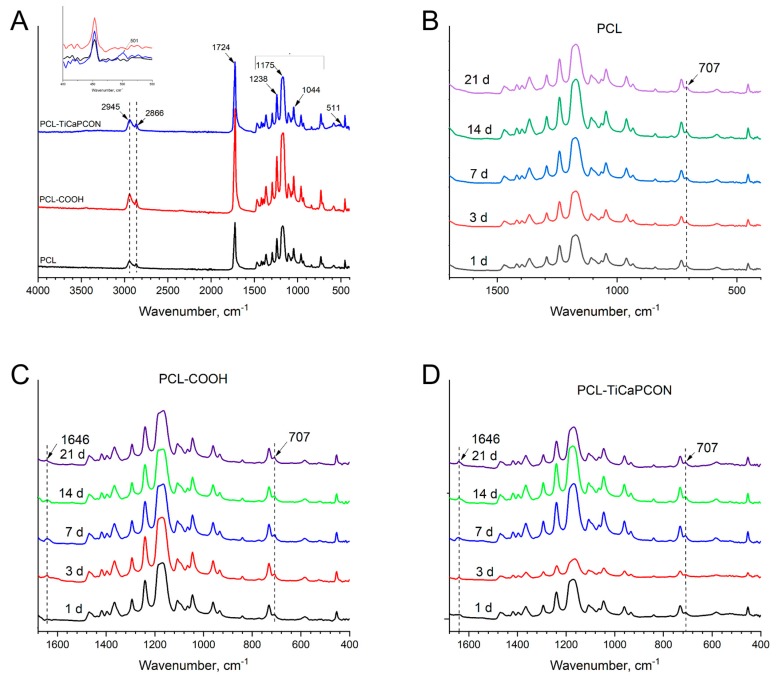
Attenuated total reflection–Fourier transform infrared (ATR–FTIR) spectra of PCL, PCL–COOH, and PCL–TiCaPCON before (**A**) and after immersion in SBF (**B**–**D**).

The attenuated total reflection (ATR) FTIR spectra of PCL-ref, PCL–COOH, and PCL–TiCaPCON samples before immersion in SBF solution are presented in Figure 6a. The presence of PCL polymer is evidenced by the CH_2_ peaks located at 2945 and 2866 cm^−1^, and the sharp bands of C=O, C–O, and CO–O–CO located at 1724, 1175  and 1044 cm^−1^, respectively [29]. The high absorbance observed in the ranges of 1420–1400 and 900–690 cm^−1^ was attributed to the CH_2_, CH_3_, and CH bending vibrations. The strong stretching bands due to the asymmetric and symmetric C–C(=O)–O vibrations were noted between 1200–1150 cm^−1^ [30]. The ATR–FTIR spectra of the coated samples were similar to that of PCL-ref. In case of PCL–COOH nanofibers this result can be explain by the fact that all chemical bonds of plasma polymer also present in the structure of PCL nanofibers. Note that after plasma treatment, the C–C(=O)–O, C–O, and C–O–C bands became broader and their intensity increased. The ATR–FTIR spectrum of the TiCaPCON-coated nanofibers revealed a small peak at 501 cm^−1^ (Figure 6a (inset)) which can be assigned to the Ti–O bonds [31]. 

Figure 6b–d compares the ATR–FTIR spectra of PCL-ref, PCL–COOH, and PCL–TiCaPCON nanofibers recorded in the range of 400–1600 cm^−1^ after immersion in the SBF for 1, 3, 7, 14, and 21 days. In case of PCL-ref sample, no Ca related peaks were observed (Figure 6b). In contrast, the ATR–FTIR spectra of PCL–COOH and PCL–TiCaPCON nanofibers exhibited significant changes after immersion in SBF (Figure 6c,d). The appearance of Ca related peak at 1646 cm^−1^ is due to Ca electrostatic conjugation through the reaction between –COO^−^ and Ca^2+^. Moreover, after the mineralization of PCL–COOH and PCL–TiCaPCON nanofibers, the weak peak at 707 cm^−1^ corresponding to the Ca–O bond [32] and the peaks at 601 and 564 cm^-1^ relating to phosphate ions were observed (Figure S1 (Supporting materials)).

The ATR–FTIR results described above were further confirmed by XPS. After immersion into SBF, the XPS analysis of PCL–COOH sample revealed the incorporation of calcium (Table 1). Note for comparison that after SBF tests, no changes in the XPS spectra of the PCL-ref sample (not shown) and no Ca on its surface were observed. In addition, the XPS C1s spectra exhibited small changes in the C(O)O and C=O contributions, apparently due to the fact that the Ca^2+^ ions coordinated with carboxyl and carbonyl groups of the PCL–COOH layer (Figure 3d,e). Considering that the concentration of Ca is rather low (~1.2 at.%) and the XPS method has a high surface sensitivity, it is reasonably to assume, that the minerelized layer is either very thin or contains little calcium. 

### 3.4. Cytocompatibility

**Figure 7:**
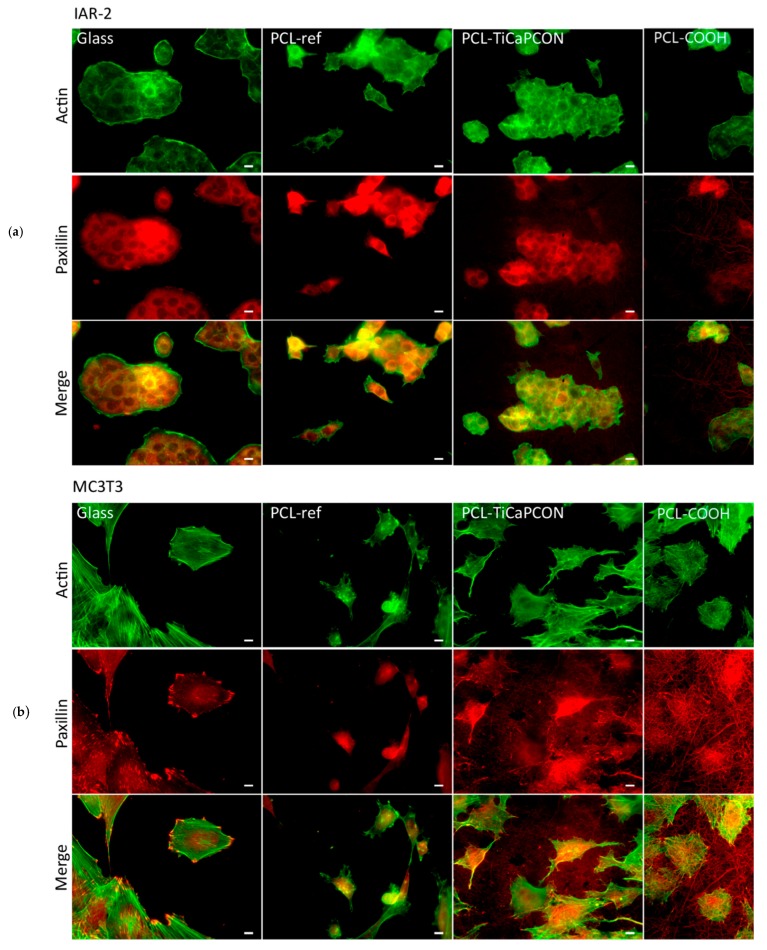
The fluorescence microscopy images showing the actin cytoskeleton and focal adhesions of IAR-2 (**a**) and MC3T3-E1 (**b**) cells on the surface of tested samples. Staining for F-actin (green) and paxillin (red). Bar is 10 μm.

**Figure 8:**
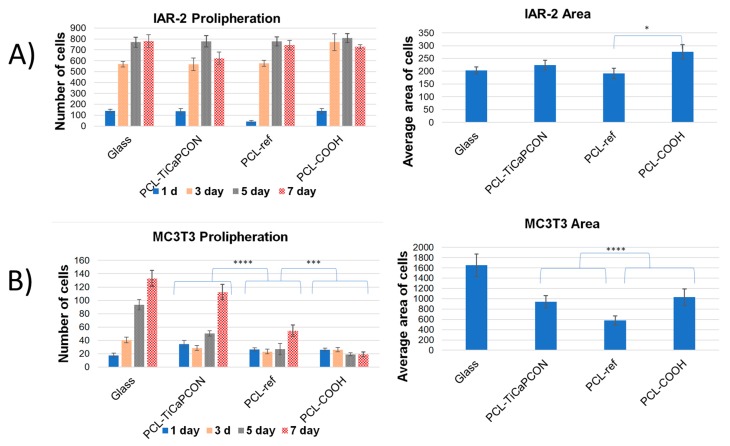
Proliferation and spreading of IAR-2 (**A**) and MC3T3-E1 (**B**) cells on the surfaces of tested samples. *—*p* < 0.05, ***—*p* < 0.001, ****—*p* < 0.0001.

In order to compare the adhesiveness of surface-modified PCL nanofibers to different types of cells, the attachment of MC3T3-E1 and IAR-2 cells was studied by fluorescence microscopy (Figure 7). The PCL-ref sample and glass coverslip were used as controls. Figure 8a compares the average area of IAR-2 cells cultivated on the surface of different substrates. The obtained results show that the surface of all tested nanofibers is adhesive for IAR-2 cells. The fluorescence microscopy images used for the quantification of cell spreading are depicted in Figure S2 (Supporting materials). As seen in Figure 8a, the COOH-modified nanofibers exhibit the largest cell area (average area of cells increased by 35% and 20% in comparison with PCL-ref and PCL–TiCaPCON). The IAR-2 cells were observed to be well spread on the surface of all tested samples. IAR-2 proliferation tests showed no statistically significant difference between the tested samples (Figure 8). Thus, PCL-ref, PCL–COOH, and PCL–TiCaPCON nanofibers are cytocompatible. 

In case of the MC3T3-E1 cells, the difference between the samples becomes more noticeable and less clear. Although the average area of MC3T3-E1 cells cultivated on the surface of polymers was notably reduced in comparison with glass control (Figure 8), the actin cytoskeleton organization was quite different. The PCL-ref sample revealed single immature spherical cells and vast areas not occupied by osteoblastic cells. In contrast, the fluorescent microscope images presented in Figure 7a indicate that the MC3T3-E1 cells well spread on the surface of PCL–COOH and PCL–TiCaPCON substrates. This indicates that the surfaces of COOH plasma polymerized and TiCaPCON-coated PCL are adhesive for the MC3T3-E1 cells. 

The proliferation of MC3T3-E1 cells on the surface of PCL-ref and PCL–COOH nanofibers was lower in comparison with the glass control (Figure 8). At the same time, a high cell proliferation activity was observed in case of PCL–TiCaPCON sample. After 7 days, the number of cells on the surfaces of PCL–TiCaPCON nanofibers and glass control were not statistically significant. To sum up, note that the COOH plasma surface polymerization of PCL scaffolds slightly improves the adhesion of IAR-2 cells, which is important for faster healing of soft tissues, whereas the TiCaPCON deposition contributes to better adhesion, spreading, and proliferation of the MC3T3-E1 cells, which are important characteristics for application as bone fillers. 

Finally note that the PCL surface modification also affects the nanofiber dissolution. PCL–TiCaPCON and PCL–COOH samples were observed to completely dissolve in SBF within 30 days of incubation. In contrast, only 27% of degradation was reported for PCL nanofibers with the same diameter as PCL-ref samples used in this study [27]. Accelerated nanofiber dissolution may be induced by intensive ion irradiation during the deposition of TiCaPCON film leading to structural changes in the PCL. Additionally, the extremely low WCA value of PCL–TiCaPCON indicates high affinity of water molecules to PCL–TiCaPCON nanofibers, thereby accelerating hydrolysis of hydrophilic PCL–TiCaPCON nanofibers compared to hydrophobic PCL-ref sample.

## 4. Discussion

The morphology and surface chemistry of an implant substrate influence the adhesion and proliferation of osteoblastic cells [33]. The nanofiber structure is very suitable for bone tissue engineering since it mimics the architecture of the extracellular matrix. The available literature data concerning the nanofiber types, mineralization methods and utilized cells are summarized in Table 2. COOH-functionalized PCL nanofibers have been used as an effective template to induce HA formation [28]. This is due to the capability of carboxylate ions (COO^−^) to adsorb calcium ions (Ca^2+^) and contribute to HA crystallization as a result of exposure to phosphate ions (PO_4_^3−^). Thus, the presence of carboxyl groups on the nanofiber surface induces HA formation and mineralization. Similar results for polylactic-glicolic acid (PLGA) were reported [34]. The presence of calcium layer or HA structures favors the adhesion and proliferation of various stem and/or osteoblastic cells. However, the grafting of COOH groups solely is not an efficient tool. As shown here and in a number of previous publications, the formation of a Ca-based layer requires exposure to the SBF for several days. This approach is not attractive for large-scale application. 

It is well known that wettability and surface topography are two important surface parameters determining the cell/material interaction [35]. It is usually assumed that cells adhere and proliferate well on the hydrophilic surfaces. However, the wettability itself depends on both material chemistry and topography. The effect of polymer surface topography on cell proliferation has been studied previously. For example, argon/oxygen plasma was used to modify the hydrophobic surfaces of polystyrene samples [34]. Although sample wettability was improved by treatments of both plasma types (lower WCA values were observed after oxygen plasma treatment), only oxygen plasma processing led to notable changes in surface roughness. Better adhesion and proliferation of unrestricted somatic stem cells on the surface of oxygen plasma-treated samples can be explained by improved hydrophilicity, higher surface roughness, and specific surface chemistry (the presence of hydroxyl and carboxyl groups on the surface). 

The surface of unmodified PCL-ref nanofibers used in the present study was relatively smooth and hydrophobic. Their surface topography was not noticeably changed after the SBF tests. In contrast, due to mineralization, the hydrophilic surfaces of PCL–COOH and PCL–TiCaPCON samples become formless (Figure 2g). Considering, that the topography of PCL–COOH and PCL–TiCaPCON nanofibers after the SBF tests is very similar, it is reasonable to assume that the difference in the MC3T3 adhesion and proliferation on their surface is more related to surface chemistry. This is consistent with the results by Keshel et al. [34]. Since the PCL–TiCaPCON sample is more hydrophilic compared to the PCL–COOH counterpart, the relative density of polar groups on its surface should be higher. 

In this study, PCL, PCL–COOH, and PCL–TiCaPCON nanofiber to cell interactions were carefully evaluated using two types of cells (MC3T3-E1 and IAR-2). The selected MC3T3 is an osteoblast precursor cell line derived from mouse calvaria [36]. Our approach has shown that the deposition of TiCaPCON layer improves the adhesion and proliferation of osteoblastic cells and promotes the Ca-based mineralized layer formation. Regarding MC3T3-E1 cells, nanofibers with TiCaPCON layer are more effective compared to COOH-immobilized PCL. Special surface chemistry and improved wettability of PCL–TiCaPCON nanofibers enhanced the adhesion of MC3T3-E1 cells. PCL–COOH samples showed high Ca adsorption during the SBF tests, but the MC3T3-E1 cell proliferation on their surfaces was low. 

In contrast, PCL–COOH sample have shown better adhesion and proliferation of IAR-2 cells. Hence, the surface functionalization strategy should be selected for specific applications carefully. Finally note that the plasma treatment of PCL nanofibers not only improves their wettability, but also increases the number of carboxylic groups and therefore affects the surface charge, which can be used for further surface modification and fabrication of PCL-based nanohybrids. 

## 5. Conclusions

Biodegradable PCL nanofibers were subjected to different surface modifications to evaluate the material/cell interactions and the mineralization ability in vitro. The results indicated that COOH surface plasma polymerization and TiCaPCON thin film deposition significantly improve the biocompatibility of PCL nanofibers. However, different methods of surface modification led to different osteoblastic and epithelial cell responses. Deposition of a thin TiCaPCON film resulted in improved adhesion and proliferation of MC3T3-E1 cells compared with COOH-modified PCL nanofibers. In addition, the TiCaPCON layer promoted the growth of Ca-based mineralized layer in simulated body fluid (1×). Thus, PCL–TiCaPCON nanofibers can be considered as promising material for replacing damaged bone tissue and/or healing of bone defects. In contrast, the enhanced adhesion and proliferation of IAR-2 cells was observed on the surface of PCL–COOH nanofibers, hereby indicating their high potential for skin reparation and wound dressing. Therefore, for a specific application, the surface modification method must carefully be selected. 

## Figures and Tables

**Table 1:** Atomic percentages of the elements determined by X-ray photoelectron spectroscopy (XPS) analysis.

Sample Name	(C), at.%	(O), at.%	(N), at.%	(Ca), at.%	(Na), at.%	(Ti), at.%
PCL-ref	76.0	24.0	0.0	0.0	0.0	0.0
PCL–TiCaPCON	55.4	22.5	8.6	1.1	0.0	8.4
PCL–COOH	77.0	23.0	0.0	0.0	0.0	0.0
PCL–COOH-12h	75.0	22.0	1.1	1.5	0.4	0.0
PCL–COOH-24h	77.3	20.1	1.0	1.2	0.3	0.0
PCL–COOH-72h	78.2	18.4	2.4	1.0	0.0	0.0

**Table 2:** The effect of mineralization method on the cell/nanofiber interaction.

Nanofiber Chemical Composition	Mineralization Method	Cell Type	Cell/Material Interaction	Reference
PCL/gelatin, PCL/gelatin/nano–hydroxyapatite (HA)	nano-HA-doped electrospun solution	DPSCs	Cell attachment and growth was not improved	[37]
PCL–COOH PCL–COOH-SBF	SBF solution	hASCs	Improved osteogenic differentiation of SBF-treated samples	[38]
PLA PLA/Ca_3_(PO_4_)_2_ PLA/Ca_3_(PO_4_)_2_/BSA	SBF-solution, deposition of amorphous Ca_3_(PO_4_)_2_ film	MG-63	No significant differences for cell adhesion and spreading	[39]
PLA/TSF, PLA/TSF/HA	1.5× SBF doped with 1 wt% of asparaginic acid	MSCs	Improved cell proliferation and differentiation on the PLA/TSF/HA	[14]
PLGA, PLGA–COOH, PLGA–COOH-Glu_6_	1.5× SBF	hMSCs	Improved proliferation	[34]
PCL/nano–HA PCL/nano–HA–COOH	nano-HAp-doped electrospun solution	MC3T3-E1	Enhanced attachment, proliferation, and differentiation	[29]
PCL, PCL–COOH PCL–COOH/PRP	-	MRC-5	The number of adhered cells did not differ significantly. More uniform cell distribution on the surface of PCL–COOH	[40]
PLGA (control), PLGA/HA PLGA/HA/ Dexamethasone	nano-HA-doped electrospun solution	MC3T3-E1	Slow cell proliferation compared with control	[41]
PLLA/PCL	Electrochemical deposition	rBMSCs	enhanced the osteogenic differen- tiation and proliferation of rBMSCs for treated sample	[17]
BR or G-BR nanoparticles into PHBV nanofibers	Electrochemical deposition	hFOB	Enhanced cell proliferation	[42]

Dental pulp stem cells (DPSCs), human adipose derived stem cells (hASCs), mesenchemal stem cells (MSCs), rate bone MSCs (rBMSCs), human fetal osteoblastic cells (hFOB).

## Supplementary Materials

The following are available online at https://www.mdpi.com/2079-4991/9/12/1769/s1, Figure S1: The comparison of PCL-ref-SBF, PCL-COOH-SBF and PCL-TiCaPCON-SBF after immersion into SBF for 7 days for identification of phosphate ions peaks, Figure S2: The example of the images for demonstration of the cell quantification.
