# Supplementary material for: Comparison of Different Approaches to Surface Functionalization of Biodegradable Polycaprolactone Scaffolds
Source: Nanomaterials (Basel). 2019 Dec 12;9(12):1769. doi: 10.3390/nano9121769 (PMC6955782; doi:10.3390/nano9121769)
Supplement: Supplementary file 1 [file nanomaterials-09-01769-s001.pdf]

nanomaterials

Article

Comparison of Different Approaches to Surface Functionalization of Biodegradable Polycaprolactone Scaffolds

Elizaveta S. Permyakova [1], Philipp V. Kiryukhantsev-Korneev [1], Kristina Yu. Gudz [1], Anton S. Konopatsky [1], Josef Polčak, [2,3] Irina Y. Zhitnyak [4], Natalia A. Gloushankova [4], D. V. Shtansky [1] and Anton M. Manakhov [5,*]

1 National University of Science and Technology "MISIS", Leninsky prospect 4, Moscow, 119049, Russia permyakova.elizaveta@gmail.com (E.S.P.); kiruhancev-korneev@yandex.ru (P.V.K-K.); kristinkagudz@mail.ru (K.Y.G.); konopatskiy@misis.ru (A.S.K.); shtansky@shs.misis.ru (D.V.S.)
2 CEITEC—Central European Institute of Technology, Brno University of Technology, Purkyňova 123, Brno, Czech Republic; polcak@fme.vutbr.cz
3 Institute of Physical Engineering, Brno University of Technology, Technicka 2896/2, 616 69 Brno, Czech Republic
4 N.N. Blokhin Russian Cancer Research Center, Kashirskoe shosse 24, Moscow 115478, Russia; irishaz@mail.ru (I.Y.Z); natglu@hotmail.com (N.A.G)
5 Scientific Institute of Clinical and Experimental Lymphology– Branch of the ICG SB RAS, 2 Timakova str., 630060 Novosibirsk, Russian Federation
* Correspondence: ant-manahov@ya.ru; Tel.: +7-915-8494059

Content:

1) Additional FTIR spectra
2) Microscope image for cell quantification analysis

1) Additional FTIR spectra

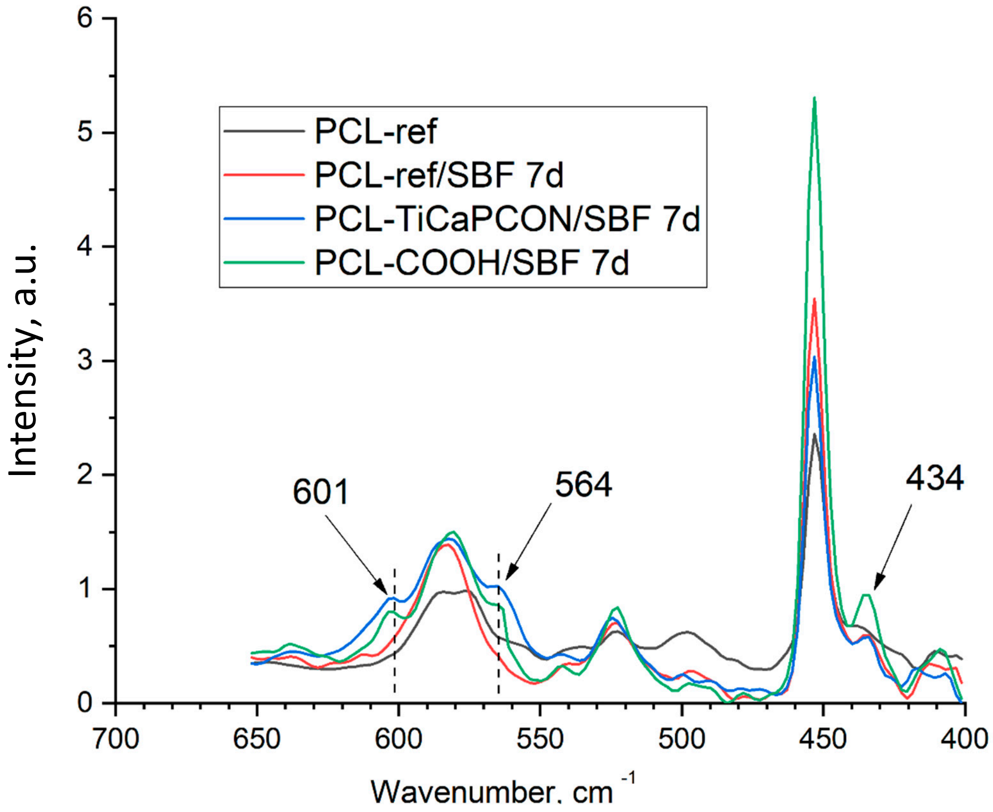

Figure S1. The comparison of PCL-ref-SBF, PCL-COOH-SBF and PCL-TiCaPCON-SBF after immersion into SBF for 7 days for identification of phosphate ions peaks.

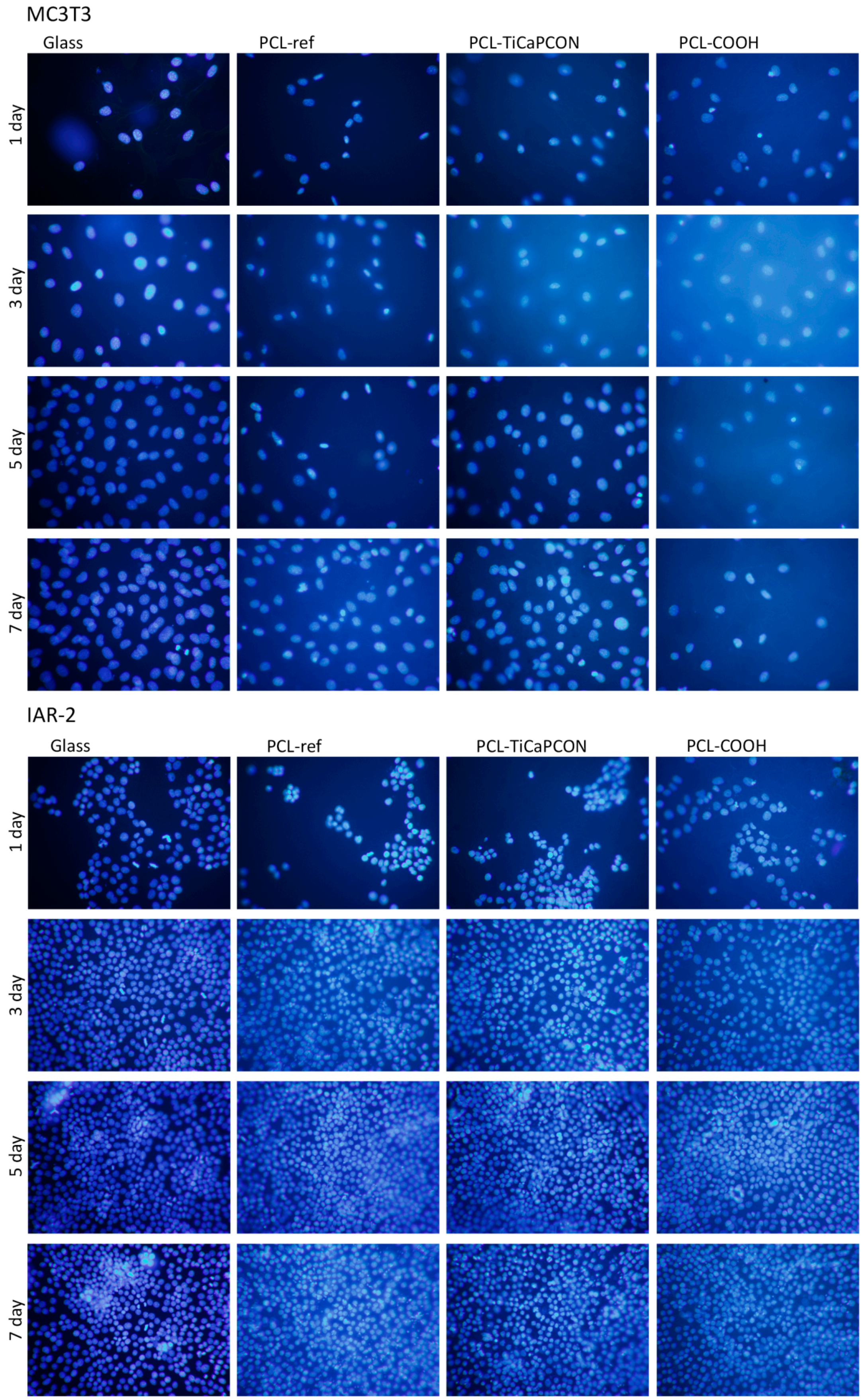

Figure S2. The example of the images for demonstration of the cell quantification.
